# Exploiting Interdata Relationships in Prostate Cancer Proteomes: Clinical Significance of HO-1, HSPB1, DDB1 and 14-3-3ζ/δ Interaction

**DOI:** 10.1200/GO.22.52000

**Published:** 2022-05-05

**Authors:** Sofia Lage-Vickers, Pablo Sanchis, Juan Bizzotto, Ayelen Toro, Maria P. Valacco, Javier Cotignola, Elba Vazquez, Geraldine Gueron

**Affiliations:** ^1^ IQUIBICEN CONICET, Buenos Aires, Argentina

## Abstract

Prostate cancer (PCa) is the second most common type of cancer in men and the sixth leading cause of cancer-related death in men worldwide. The discovery of new therapeutic avenues in PCa, and the development of effective drugs in the era of personalized medicine, would greatly benefit from the field of proteomics. We have previously shown the anti-tumoral role of Heme oxygenase 1 (HO-1) in PCa.

**METHODS:**

In this work, we undertook a mass spectrometry-based proteomics study to assess whether in PCa cells and under oxidative stress conditions, HO-1 could interact with proteins previously documented to have nuclear localization.

**RESULTS:**

Among the HO-1 interactors, we identified 11 proteins with nuclear localization. Significant and positive correlation between HMOX1 and six of those genes was observed, using the GSE70770 dataset. Alternatively, HMOX1 and YWHAZ showed negative correlation. High expression of HNRNPA2B1, HSPB1, NPM1, DDB1, HMGA1, ZC3HAV1, and HMOX1 was associated with an increased relapse-free survival (RFS) in PCa patients through univariable analyses. Further, PCa patients with high HSPB1/HMOX1, DDB1/HMOX1, and YWHAZ/HMOX1 showed a worse RFS compared with patients with lower ratios. Moreover, by using a prognostic risk score model, a decrease in RFS for patients with higher scores of this signature was observed. However, the only factor significantly associated with a higher risk of relapse was high YWHAZ. HSPB1, DDB1 and YWHAZ independence from PCa clinic-pathological parameters was confirmed through multivariable analyses. Moreover, co-immunoprecipitation analysis in PCa cells ascertained HO-1/14-3-3ζ/δ (YWHAZ encoding gene) interaction.

**CONCLUSION:**

Herein, we report novel interactions between HO-1 and HSPB1, DDB1 and 14-3-3ζ/δ, highlighting their clinical relevance in PCa.

